# *Mycoplasma fermentans* Inhibits the Activity of Cellular DNA Topoisomerase I by Activation of PARP1 and Alters the Efficacy of Its Anti-Cancer Inhibitor

**DOI:** 10.1371/journal.pone.0072377

**Published:** 2013-08-27

**Authors:** Reuven Afriat, Shulamith Horowitz, Esther Priel

**Affiliations:** The Shraga Segal Department of Microbiology, Immunology & Genetics, Faculty of Health Sciences, and the Soroka University Medical Center, Ben-Gurion University, Beer-Sheva, Israel; Wayne State University School of Medicine, United States of America

## Abstract

To understand the effects of the interaction between Mycoplasma and cells on the host cellular function, it is important to elucidate the influences of infection of cells with Mycoplasma on nuclear enzymes such as DNA Topoisomerase type I (Topo I). Human Topo I participates in DNA transaction processes and is the target of anti-cancer drugs, the camptothecins (CPTs). Here we investigated the mechanism by which infection of human tumor cells with *Mycoplasma fermentans* affects the activity and expression of cellular Topo I, and the anti-cancer efficacy of CPT. Human cancer cells were infected or treated with live or sonicated *M. fermentans* and the activity and expression of Topo I was determined. *M. fermentans* significantly reduced (by 80%) Topo I activity in the infected/treated tumor cells without affecting the level of Topo I protein. We demonstrate that this reduction in enzyme activity resulted from ADP-ribosylation of the Topo I protein by Poly-ADP-ribose polymerase (PARP-1). In addition, pERK was activated as a result of the induction of the MAPK signal transduction pathway by *M. fermentans*. Since PARP-1 was shown to be activated by pERK, we concluded that *M. fermentans* modified the cellular Topo I activity by activation of PARP-I via the induction of the MAPK signal transduction pathway. Moreover, the infection of tumor cells with *M. fermentans* diminished the inhibitory effect of CPT. The results of this study suggest that modification of Topo I activity by *M. fermentans* may alter cellular gene expression and the response of tumor cells to Topo I inhibitors, influencing the anti-cancer capacity of Topo I antagonists.

## Introduction

Mycoplasmas, which belong to the Mollicutes class, are the smallest self-replicating eubacteria, devoid of a cell wall and surrounded only by a plasma membrane. Their small genome size (ranging from 580 to 1380 kbp) results in limited metabolic capabilities and parasitism [1], [2]. Mycoplasmas can be found as parasites in a wide range of hosts including humans, animals, insects, plants, and cells grown in tissue culture.

In humans, some Mycoplasma species are found as commensal inhabitants, while other were shown to be associated with infectious diseases and post-infection pathologies [3], [4].

Most of the known Mycoplasma species are found as membrane surface parasites, and recently, some were shown to enter the cells and become intracellular residents [5].

Mycoplasma may cause chronic infections due to sophisticated mechanisms for evasion from immune surveillance (i.e., molecular mimicry, a unique type of antigenic variation), up-regulating or down-regulating cytokine secretion, adhesion molecules expression, transcription factors expression, MAP kinases activity, apoptotic pathways, and more [2], [3].

Recently, many reports have strongly supported the ability of Mycoplasma to cause or promote oncogenic transformation [6]–[9], and the search for the link between Mycoplasma and cancer is currently being explored [10].

The lipoproteins (LPMf) of *Mycoplasma fermentans*, a human pathogen, was thoroughly investigated during the last decade. LPMf was shown to alter the functions of the immune system cells by inducing expression of oncogenes [1], affecting several factors participating in signal transduction proteins [11]–[13], transcription [14], and the apoptotic process [11], [15], [16]. *M. fermentans* was shown to inhibit the apoptosis process induced by tumor necrosis factor α (TNFα) [17], [18]. All these led to the assumption that infection of tumor cells by Mycoplasma may affect the activity and expression of essential nuclear enzymes such as topoisomerases, which are the targets of several anti-cancer drugs and thus interfere with the anti-cancer efficacy of these drugs. DNA topoisomerases are a family of essential nuclear enzymes that are responsible for controlling the topological state of the DNA molecules. They participate in most DNA transactions such as replication, transcription, recombination, and chromatin remodeling [19]–[21]. DNA topoisomerases are classified as either type I (cleaves one strand of DNA) or type II (cleaves two strands of DNA). Both enzyme types are further categorized into subgroups according to structural and functional features. Members of each family of enzymes are distinct in sequence, structure, and functions [22].

The catalytic activity of DNA topoisomerases involves the formation of transient covalent bridges of enzyme-DNA complexes. A tyrosyl group in the active site of the enzyme attacks a phosphodiester bond on the DNA backbone and remains covalently attached to one side of the break, leaving an opposite free hydroxyl (OH) end that allows the religation step, after DNA topology is resolved, by a second nucleophilic attack of the covalent enzyme-DNA phosphotyrosine bond, releasing the enzyme for the next catalytic cycle. The involvement of these enzymes in essential cellular processes tagged topoisomerases as important targets for anti-cancer treatments and for the development of potent, more effective, anticancer drugs [22], [23]. The cytotoxicity of Topoisomerases inhibitors such as Camptothecin (CPT) and its derivatives TPT and CPT-11 (which are approved for clinical use), stems from their ability to stabilize the cleavable complex of Topo–DNA, which introduces single and double strand breaks in the DNA [21], [24], [25]. Topoisomerase activity is influenced by several post-translational modifications, among them phosphorylation, poly-ADP-ribosylation, and ubiquitination. Recent work done in our laboratory demonstrated the O-GlcNAcylation of Topo IB, which affects its activity [26].

The phosphorylation of DNA topoisomerase I by casein kinase II (CK II) and protein kinase C (PKC) up-regulate the enzyme DNA relaxation activity, whereas dephosphorylation by alkaline phosphatase inhibited this activity. In addition, poly-ADP ribosylation by poly-ADP ribose polymerase (PARP-1) of the enzyme protein was found to down-regulate its activity [20], [27]. PARP-1 is known to be activated by DNA breaks; however recently, it was reported that PARP-1 can be activated by phosphorylated ERK2 in the absence of stress conditions or DNA damage [28]. In recent studies Mycoplasma was demonstrated to be capable of activating various MAPKs, such as SAPK/JNK, p38, NF-kB, AP-1, and ERK 1/2 in response to Mycoplasma-derived membrane lipoproteins [11], [29]–[31]. Thus it is important to investigate the possibility that the cellular Topo I and the efficacy of CPTs as anti-cancer agents might be affected by Mycoplasma infection.

## Materials and Methods

### Cells

Human breast cancer cell lines -MCF7 (American Type Culture Collection, HTB-22) and human glioblastoma cells- U251 (HTB-17) kindly received from Porgador A, Ben-Gurion University. Cells were cultured as monolayers in DMEM and RPMI 1640 medium (Biological Industries, Beith Haemek, Israel), respectively, supplemented with 10% fetal bovine serum, 50 IU penicillin, 50 µg/ml streptomycin, and L-glutamine (0.29 µγ/ml). Cell lines were grown in a humidified incubator supplemented with 5% CO_2_ at 37°C.

### Enzymes, Antibodies, and Compounds

Stock solutions of Camptothecin (Sigma, Israel) at 20 mM (dissolved in 100% DMSO) were stored in aliquots at −70°C and diluted in DMSO before being added to the reaction mixture or to the cell culture medium. Supercoiled DNA plasmid pUC19 was purchased from MBI Fermentas (Hanover, MD, USA). PD98059 and 3-aminobenzamide (3AB) were purchased from Sigma-Aldrich (Rehovot, Israel).

The primary antisera were as follows: monoclonal mouse anti-β-actin antibody (MP Biomedicals, LLC); goat polyclonal IgG (C-15) anti-topo I, mouse monoclonal IgG antiphospho-ERK (E-4), and rabbit polyclonal IgG anti-ERK (C-16) (Santa Cruz Biotechnology Inc., CA, USA); mouse monoclonal anti-poly-ADP ribose antibodies, (Serotec, Oxford, UK); and goat anti-mouse and anti-rabbit IgG second antibodies (Biolabs Inc., Ipswich, MA, USA). For immunoprecipitation assays, anti-Topo I antibody derived from scleroderma patient serum was used (TopoGene, Florida, USA).

Enhanced chemiluminescence (ECL) reagents were purchased from Biological Industries (Beit Haemek, Israel).

### Mycoplasma Growth

*M. fermentans strain* K7 was cultured in SP4 broth at 37°C and 5% CO_2_ until log phase. One ml aliquots containing 2×10^8^ colony-forming units (CFU) were kept frozen at −70°C until used. For each experiment, one aliquot was thawed, cultivated in SP4 broth at a dilution of 1/10 for 24 h (37°C, 5% CO_2_), followed by further dilution of 1/50 until a log phase was observed (after approx. 24 hrs). The exact quantity of *M. fermentans* was determined by counting CFUs, as previously described [32], [33].

### Mycoplasmal Protein Extract Preparation

*M. fermentans* was cultivated as above; the 500 ml culture was pelleted (13000×g, 30 min at 4°C) and washed twice with phosphate-buffered saline (PBS). The pellet was then re-suspended in PBS. CFU/ml was determined, and the re-suspended pellet was stored at −70°C for further experiments. Frozen pellets of *M. fermentans* were thawed and sonicated at 4°C for 4×30 sec at 80% power (50% duty cycle; Heat Systems Ultrasonics, Inc.) in the presence of the protease inhibitor phenylmethylsulphonyl fluoride (PMSF; 0.001 M). These conditions of sonication resulted in a non-live mycoplasmal preparation (no growth was observed in repeated culturing procedures). Protein concentration of the sonicated *M. fermentans* was determined by the Bio-Rad protein assay kit (Richmond, CA); 1×10^8^ CFU corresponded to 10 µg mycoplasmal protein.

### Treatment of Malignant Cell Lines with Live *M.* *fermentans* or *M. fermentans* Total Protein

MCF7 and U251 cells, prewashed once with PBS (1200 RPM, 5 min at 4°C) and re-suspended in a new culture medium at a concentration of 2×10^5^ cells/ml, were cultured in 96-well sterile plates (Corning Inc., Corning, NY) at a final concentration of 4×10^4^/200 µl/well. Live *M. fermentans* at log phase, prewashed once in PBS (13000×g, 30 min at 4°C), and re-suspended in RPMI 1640 medium containing 10% inactivated FCS, 1% penicillin, and 1% glutamine, were added to the cell cultures at MOI 1000∶1 (4×10^7^ CFU/well). The co-cultures were incubated for five hrs at 37°C, 5% CO_2_. For experiments with *M. fermentans* total protein, a concentration of 20 µg/ml (corresponding to 2×10^8^ CFU) was added to the examined tumor cells (2×10^5^ cells/ml) for 24 hrs at 37°C, 5% CO_2_.

### DNA Amplification of *M. fermentans* by Reverse Transcription-PCR (RT-PCR) Using a Nucleotide Sequence within the Insertion Sequence-Like Element

Total RNA was extracted from the infected MCF7 and U251 cells using TRI Reagent (T9424; Sigma-Aldrich, Rehovot, Israel), then reverse transcribed using 1 µg of total RNA from the examined cells with Oligo-dT primer, using RevertAid Kit (K1621; Fermentas, Vilnius, Lithuania). Various cDNA isolated from *M. fermentans*-infected cells for different intervals (1.5, 3, 6, 12, and 24 hrs) were amplified using REDTaq Ready Mix (R2523; Sigma-Aldrich, Rehovot, Israel). Sequences of the synthetic oligonucleotide primer pair (RWO04 and RWO05) used for specific DNA amplification of *M. fermentans* and their positions in the Insertion Sequence-Like Element (IS-like element) were previously described [34]. The primer sequences and sizes of the PCR products were as follows: RW004 Primer (24 nt): 5′GGA CTA TTG TCT AAA CAA TTT CCC and RW005 Primer (24 nt): 5′GGT TAT TCG ATT TCT AAA TCG CCT. The size of the diagnostic DNA band is 206 bp. The thermal cycling profile of amplification comprised 40 cycles at 95°C for 30 seconds (240 seconds in the first cycle), 62°C for 60 seconds, and 72°C for 60 seconds (increased to 10 minutes for the final cycle). The PCR products were visualized on a 1% agarose gel stained with ethidium bromide.

### Nuclear Protein Extracts Preparation

Nuclear extracts for Topoisomerase assays and Western blot analysis from the MCF7 and U251 cells were prepared as previously described [27], [35]–[40] and a mixture of protease inhibitors (final concentrations: 2 µg/ml aprotinin, 2 µg/ml leupeptin, 1 µg/ml pepstatin A, 2 µg/ml antipain, 100 µg/ml PMSF) was added to the extraction buffers. Total protein concentration was determined using the BIO-Rad protein assay kit (Bio-Rad Lab, CA, USA).

### Determination of the Level of Topo I Protein by Western Blot Analysis

Equal quantities of nuclear proteins derived from Mycoplasma treated or untreated MCF7 and U251 cells, were analyzed by Western blot analysis using either anti-Topo I antibody (Santa Cruz Biotechnology Inc.) or anti-β-actin antibodies (MP Biomedicals, LLC) as previously described [36], [39], [41]. The immunocomplexes were detected by enhanced chemiluminescence (ECL).

### Topo I Assay

Topo I assay was performed as previously described [26], [37]. Increasing concentrations of nuclear proteins were added to a Topo I reaction mixture containing, at a final volume of 25 µL: 20 mM Tris–HCl (pH 8.1), 1 mM Dithiothreitol, 20 mM KCl, 10 mM MγCl_2_, 1 mM ethylenediaminetetraacetic acid (EDTA), 30 µg/mL bovine serum albumin, and 250 ng pUC19 supercoiled DNA plasmid (MBI; Fermentas, Hanover, MD). Following incubation at 37°C for 30 min, the reaction was terminated by adding 5 µL of stopping buffer (final concentration: 1% sodium dodecyl sulfate, 15% glycerol, 0.5% bromophenol blue, and 50 mM EDTA, pH 8). The reaction products were analyzed by electrophoresis on a 1% agarose gel using Tris/borate/EDTA buffer (89 mM Tris–HCl, 89 mM boric acid, and 62 mM EDTA) at 1 V/cm, stained by ethidium bromide (1 µg/mL) and photographed using a short-wavelength UV lamp (ChemiImager™ 5500 equipment, Alpha Inotech Corporation, San Leandro, CA). Densitometric analysis of the results were performed with the EZ-Quant-Gel analysis software (EZ-Quant, Rehovot, Israel), and the percentage of Topo I activity was calculated using the following equation: (1 − (sample/control)) × 100 [37].

### Immunoprecipitation Assay

Mouse monoclonal anti-poly-ADP ribose (PAR) antibody (MCA 1480) was purchased from Serotec (Oxford, UK). Equal amounts of nuclear proteins (200 µg), which were pre-boiled for 5 min, were subjected to immunoprecipitation with anti-human Topo I antibody (SC) at a final volume of 100 µL of nuclear buffer (10 mM Tris–HCl, pH 7.4, 10 mM NaCl, 1.5 mM MgCl_2_, and protease inhibitors: 2 µg/mL aprotinin, 2 µg/mL leupeptin, 1 µg/mL pepstatin A, 2 µg/mL antipain, 100 µg/mL PMSF). The mixture was rotated at 4°C overnight. Protein A-Sepharose (0.1 g/mL) in TE buffer (10 mM Tris–HCl, pH 8, 1 mM EDTA) was added for an additional 1 h. The samples were centrifuged at 10,000×g for 2 min and the beads were washed three times with TE buffer. The pellet was resuspended in 25 µL of sample buffer (final concentration: 7.5% glycerol, 1% SDS, 50 mM Tris–HCl, pH 6.8, 2.5% β-mercaptoethanol, and 0.025% bromophenol blue), boiled for 5 min, and centrifuged. The samples were loaded on 7.5% SDS–PAGE, and Western blot analysis was performed using goat polyclonal IgG (C-15) anti-Topo I or anti-poly-ADP ribose (PAR) antibodies.

### Stripping of Antibodies from the Nitrocellulose Membrane

The membrane was immersed in a stripping buffer (100 mM 2-β-mercaptoethanol, 2% SDS, and 62.5 mM Tris–HCl, pH 6.7) followed by incubation at 50°C for 30 min with occasional agitation. The membrane was washed twice with TPBS (31.25 mM Na_2_HPO4, 12.5 mM Na_2_HPO_4_, 13.7 mM NaCl, 0.1% Tween) for 10 min at room temperature.

### Cell Cytotoxicity Assay

Cells (5000/well, in triplicates) were infected with *M. fermentans* at 2×10^1^–2×10^3^ MOI for 6 hrs followed by treatment with 30 µM CPT or 0.01% DMSO for an additional 12, 24, or 36 hrs. Cell survival was examined using the Neutral Red assay [42].

### Statistical Analysis

Student *t*-test was used to determine the significance between experimental samples and controls. *P* values <0.05 were considered statistically significant (*); *P* values <0.01 (**) and <0.005 (***) were considered highly significant.

## Results

### 1. Live *M. fermentans* Inhibits the DNA Relaxation Activity of Topo I

**Figure 1:**
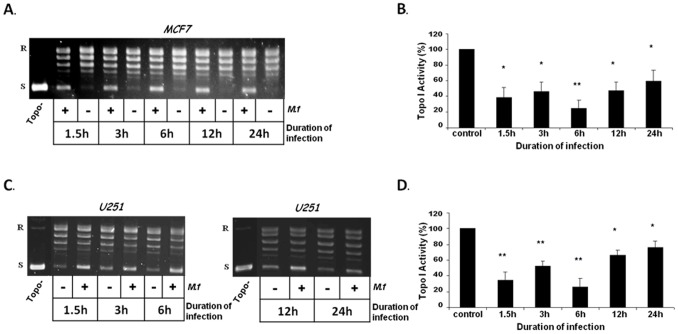
Infection of cells with live *M.* *fermentans* inhibits the DNA relaxation activity of Topo I. MCF7 (A,B) and U251 (C,D) cancer cells were infected with live *M. fermentans* (*M.f*) for various intervals at MOI of 10^3^CFU/cell. Total nuclear protein (12.5 ng) was added to a specific reaction mixture for Topo I. Reaction products were analyzed by agarose gel electrophoresis. (**A,C**) A representative picture (n = 4–5) of Topo I DNA-relaxation activity. (**B,D**) Quantification analysis of Topo I activity. Symbols: R and S are the relaxed and supercoiled form of the pUC19 DNA, respectively; Topo- no protein added to the reaction mixture. *t*-test: *p<0.05, **p<0.01, ***p<0.005.

**Figure 2:**
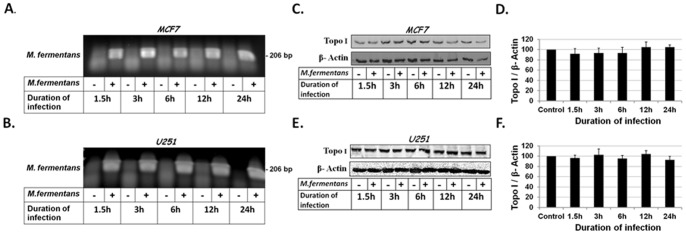
Infection of cells with live *M. fermentans* (M.f) did not alter the level of Topo I protein. Samples from the MCF7 and U251 infected cells (described in Figure 1) were analyzed for detection of mycoplasmal DNA by PCR (**A,B**). Nuclear extracts derived from MCF7 and U-251 infected cells were assayed for Topo I protein level using Western blot analysis with anti-Topo I or anti-β-actin antibodies, and quantified by densitometric analysis using the EZquant software (C–F).

The effect of live *M. fermentans* on the activity of cellular Topoisomerase I was examined in two types of cell lines: human breast cancer (MCF7) and glioblastoma (U251). Cells were infected with live *M. fermentans* at MOI of 10^3^CFU/cell, for various intervals (1.5, 3, 6, 12, and 24 hrs). Nuclear extracts were prepared from infected and uninfected cells and Topo I activity was measured. Equivalent amounts of nuclear extract proteins were added to a Topo I DNA relaxation assay mixture containing supercoiled DNA plasmid as the substrate for the enzyme; the reaction products were analyzed by agarose gel electrophoresis. Topo I activity is measured by the conversion of supercoiled plasmid to its partially or fully relaxed forms [19]. Both cell types infected by *M. fermentans* demonstrated a significant decrease, up to 80%, in the DNA relaxation activity of the enzyme, compared to uninfected cells. A gradual decrease in Topo I activity was observed starting 1.5 hr after infection with *M. fermentans*, and the peak of the reduction in the enzyme activity (80%) was detected at 6 hrs post-infection. Significant reduction in the DNA relaxation activity of Topo I was also detected 12 and 24 hrs post-infection (30–50% for MCF7, 25–40% for U-251, respectively) (Figure 1A–D). However, the degree of the reduction in Topo I activity decreased with time, suggesting that the effectiveness of the signal mediated by Mycoplasma is weakened, as was previously demonstrated for Mycoplasma-mediated signal transduction pathways [31], [43]. To confirm that the cells were actually infected and the observed effect is due to the presence of *M. fermentans*, we assayed the presence of Mycoplasma DNA in the infected cells by reverse transcriptase polymerase chain reaction (RT-PCR) assay using *M*. *fermentans-*specific primers. The results confirmed that the examined tumor cell lines were indeed infected with *M. fermentans* (Figure 2A).

To examine whether the decrease in Topo I activity in infected tumor cells is a consequence of a reduction in Topo I protein level, nuclear extracts derived from both infected and uninfected cells were analyzed by Western blot with the appropriate anti-Topo I antibodies. As depicted in Figure 2C–F, the level of Topo I protein in infected cells was similar to that found in uninfected cells. The results suggest that the reduction in Topo I activity was not due to a decrease in the enzyme protein amount.

### 2. Inhibition of the DNA Relaxation Activity of Topo I by Sonicated *M. fermentans*

**Figure 3:**
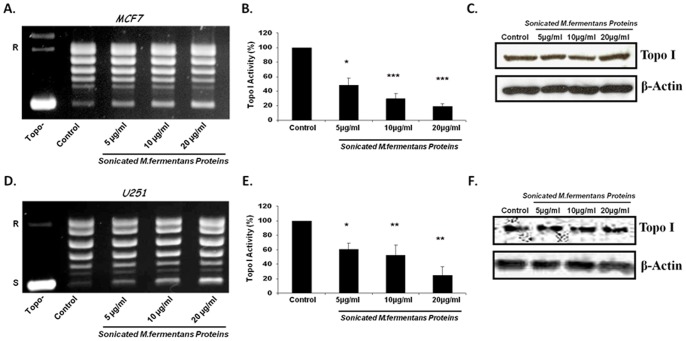
Sonicated *M.* *fermentans* Protein inhibits the DNA relaxation activity of Topo I. MCF7 (A–C) and U251 (D–F) cancer cells were treated for 24 hrs with various concentrations of *M. fermentans* proteins. Total nuclear proteins (12.5 ng) were added to a specific reaction mixture for Topo I. Reaction products were analyzed by agarose gel electrophoresis. (**A,D**) A representative picture (n = 4–5) of Topo I DNA relaxation activity. (**B,E**) Quantification analysis of Topo I activity. (**C,F**) Topo I protein level examined by Western blot analysis. Symbols: R and S are the relaxed and supercoiled form of the pUC19 DNA, respectively, Topo- no protein added to the reaction mixture. *t*-test: *p<0.05, **p<0.01, ***p<0.005.

To determine whether the Mycoplasma effect on Topo I is exerted by materials secreted from live Mycoplasma or by structural proteins of this bacteria, we examined Topo I activity in tumor cells treated with non-live, sonicated, *M. fermentans*. Cells (MCF7 and U251) were treated for 24 hrs with increasing protein concentration (5, 10, and 20 µg/ml) derived from sonicated *M. fermentans*. Nuclear extract was prepared from the treated and untreated cells and Topo I DNA relaxation activity was measured. As shown in Figure 3A,D, a significant reduction in Topo I activity was observed, in a dose-dependent manner, in cells treated with *M. fermentans* proteins (sonicate). Quantification of Topo I activity [37] from multiple experiments (n = 4) was performed. A reduction of 40–80% (*p*<0.05) in Topo I activity is observed in cells treated with 5–20 µg/ml of sonicated *M. fermentans* proteins for 24 hrs (Figure 3B,E). Although reduction of Topo I activity in cells treated with sonicated Mycoplasma was observed, the level of Topo I protein was not affected (Fig. 3C,F).

This result indicates that either live or non-live (sonicated) *M. fermentans* exerted similar effects on the cellular Topo I enzyme.

### 3. *M. fermentans* Diminished the CPT Inhibition Effect on the DNA Relaxation Activity of Topo I

**Figure 4:**
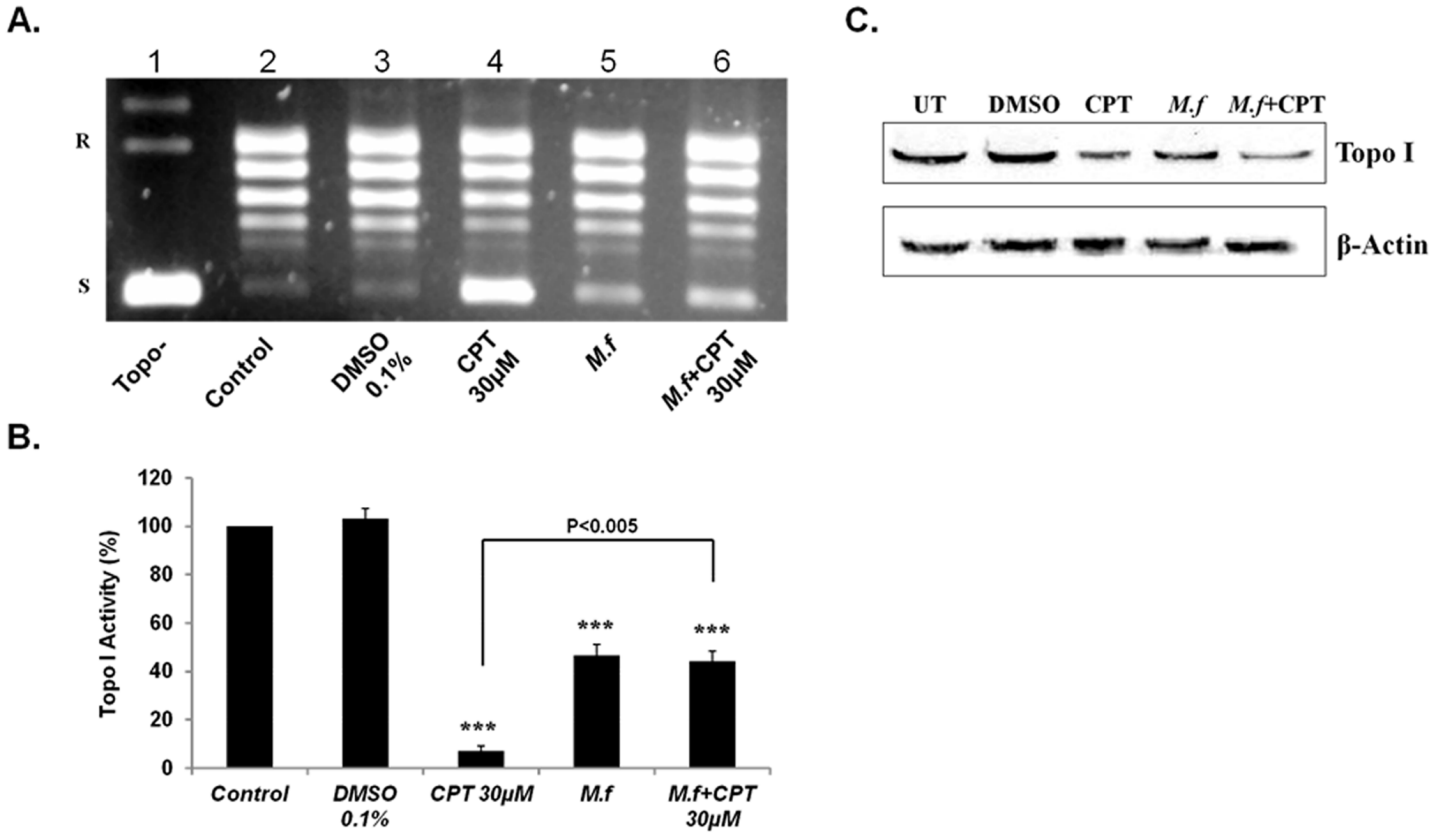
*M.* *fermentans* diminished the CPT inhibition effect on the DNA relaxation activity of Topo I. MCF7 cells were infected with *M. fermentans* (*M.f*) for 6 hrs followed by CPT (30 µM) treatments for additional 1.5 hrs. Total nuclear protein (12.5 ng) was added to a specific reaction mixture for Topo I. Reaction products were analyzed by agarose gel electrophoresis. (**A**) A representative picture n = 5, of Topo I DNA-relaxation activity. (**B**) Quantification analysis of Topo I activity. (**C**) Topo I protein level examined by Western blot analysis. Symbols: R and S are the relaxed and supercoiled form of the pUC19 DNA, respectively, Topo- no protein added to the reaction mixture *t*-test: *p<0.05, **p<0.01, ***p<0.005.

It was of interest to examine the effect of Mycoplasma infection on the inhibitory ability of known Topo I antagonists such as CPT. Initially, we conducted a series of experiments in which cells were treated with various concentrations of CPT in order to select the CPT dose that causes nearly full inhibition of the enzyme activity within a short period of time (not shown). We selected a CPT dose of 30 µM, which inhibited Topo I activity by 90–95% within 1.5 h (Figure 4A,B; compare lane 4 to lane 2). Cells were infected with live *M. fermentans* for 6 hrs at MOI of 10^3^ CFU/cell, followed by 30 µM CPT treatments for an additional 1.5 hrs. A significant diminishing in the CPT inhibitory effect (from 95% inhibition to approximately 60%) was observed in MCF7 cells exposed to the combination of *M. fermentans* infection and CPT treatment (Figure 4A,B, compare lane 6 to lane 4, *p*<0.005). Similar results were obtained with U251 cells (Figure S1 A–B). Treatment of cells with CPT is known to reduce the quantity of free Topo I protein present in the nucleoplasm due to the stabilization of the enzyme–DNA cleavable complexes by CPT. Indeed, treatment of tumor cells with CPT reduced the level of free Topo I protein (Figure 4C lane 3). The combination of Mycoplasma infection and CPT treatment did not affect the level of free Topo I protein when examined relative to the amount of β-actin protein (Figure 4C).

### 4. Poly (ADP-ribose) Polymerase (PARP) Antagonists Prevented the Mycoplasma-induced Inhibitory Effect on Topo I Activity

**Figure 5:**
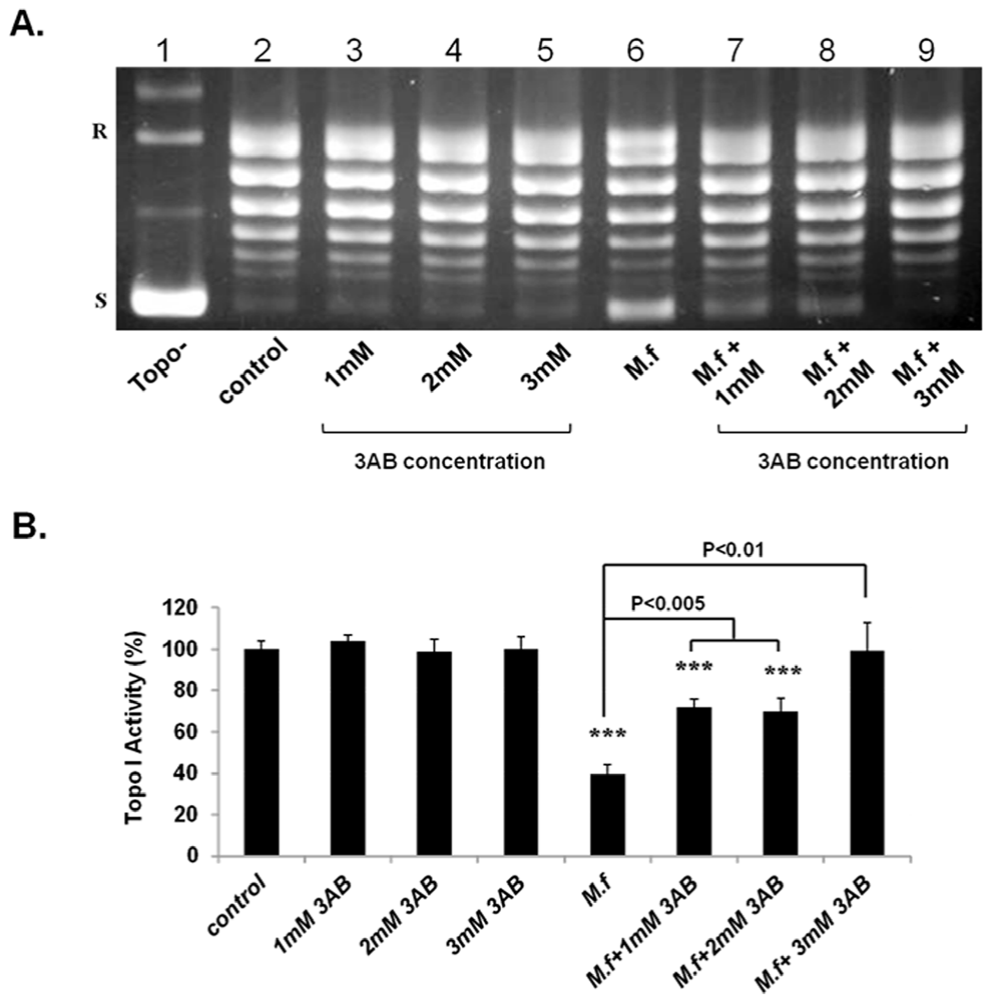
PARP inhibitor prevented the Mycoplasma-induced inhibitory effect on Topo I activity. MCF7 cells were pre-incubated with 3-aminobenzamide (3AB) for 1 hr at various concentrations followed by *M. fermentans* infection (MOI of 10^3^ CFU/cell) for an additional 6 hrs. Total nuclear protein (12.5 ng) was added to a specific reaction mixture for Topo I. Reaction products were analyzed by agarose gel electrophoresis (A) and quantification of Topo I activity was performed (B). Symbols: R and S are the relaxed and supercoiled form of the pUC19 DNA, respectively, Topo- no protein added to the reaction mixture *t*-test: *p<0.05, **p<0.01, ***p<0.005.

The aforementioned results suggest that the reduction in Topo I activity in tumor cells exerted by *M. fermentans* might be due to post-translational modifications of the enzyme proteins. Topo I undergoes several post-translational modifications, which affect its activity: Phosphorylation of Topo I by Casein kinase II or by PKC increases its activity, whereas Poly-ADP ribosylation by PARP-1 decreases its activity. In addition, ubiquitination of Topo I leads to its proteolysis [44], [45]. To determine the pathway by which *M. fermentans* affects topoisomerase I, we first investigated the effect of poly (ADP-ribose) polymerase (PARP) inhibitor –3-aminobenzamide (3AB) on Topo I activity, alone and as a pre-treatment to *M. fermentans* infection. MCF7 cells were pre-incubated with 3AB at different concentrations (1–3 mM) for 1.5 hrs followed by *M. fermentans* infection (MOI of 10^3^CFU/cell) and incubation for an additional 6 hrs. Nuclear extract was prepared and analyzed for Topo I activity as described above. The results depicted in Figure 5A–B show that pretreatment with 3AB prevented the *M. fermentans*-induced reduction in Topo I activity. Treatment of uninfected cells with 3AB for the indicated time did not influence the cellular Topo I activity (Figure 5A,B). Similar results were obtained with the U-251 cells (Figure S2 A–B).

### 5. MEK Inhibitor Prevented the Mycoplasma-induced Reduction in Topo I Activity and ERK 1/2 Phosphorylation

**Figure 6:**
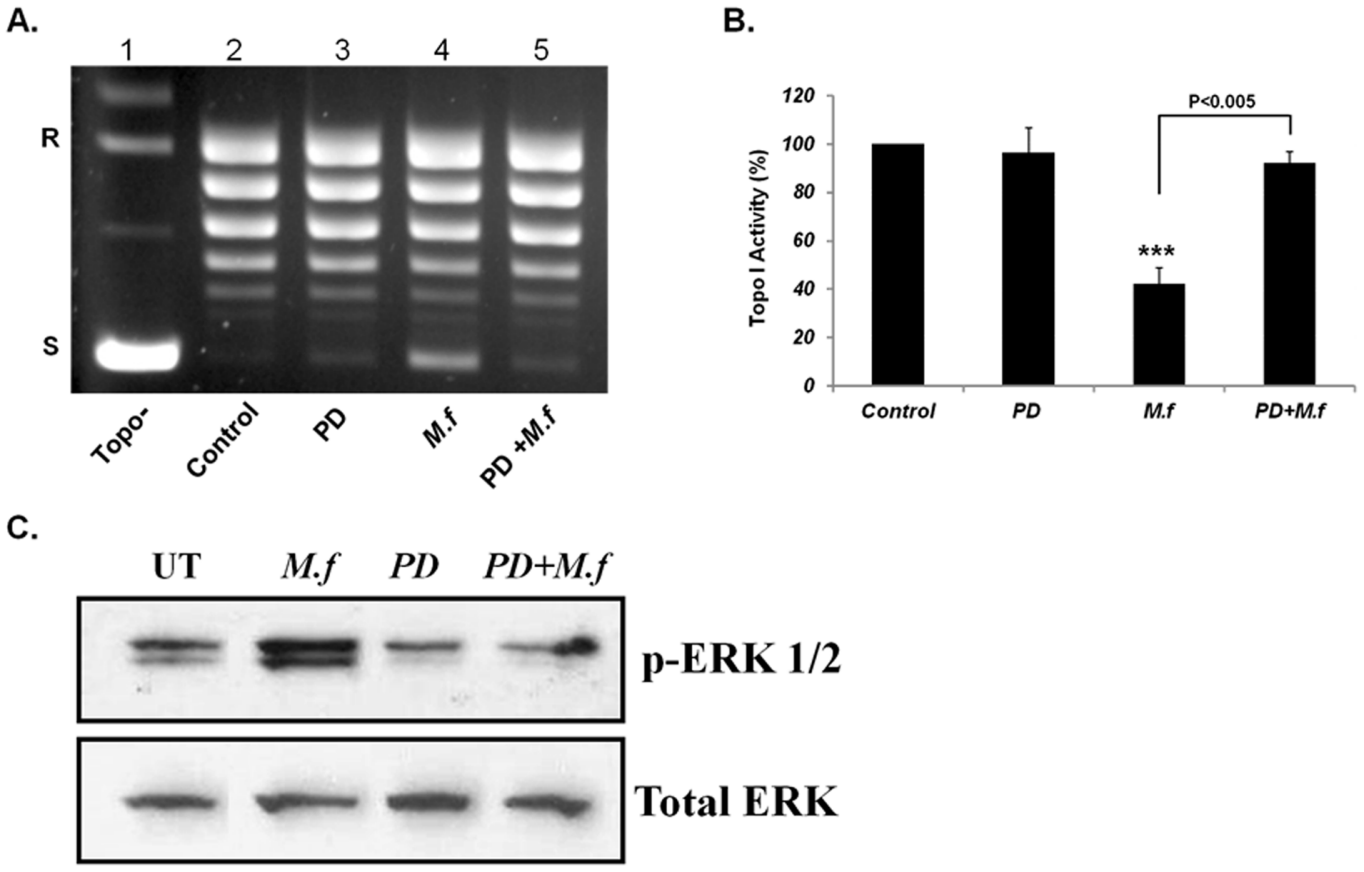
The Effect of MEK inhibitor on Mycoplasma-induced inhibitory effect on Topo I activity and on ERK 1/2 Phosphorylation in MCF7 cells. MCF7 cells were pre-incubated with MEK inhibitors (PD) for 1 hour at a concentration of 25 µM followed by *M. fermentans* infection (MOI of 10^3^CFU/cell) for an additional 6 hrs. Total nuclear protein (12.5 ng) was added to a specific reaction mixture for Topo I. Reaction products were analyzed by agarose gel electrophoresis (A) and Topo I activity was quantified (B). Phosphorylated ERK 1/2 protein level from MCF7-extract was examined by Western blot analysis (C). Symbols: R and S are the relaxed and supercoiled form of the pUC19 DNA, respectively, Topo- no protein added to the reaction mixture *t*-test: ***p<0.005.

Recent reports demonstrated that PARP-1 is phosphorylated by ERK [28]. The effect of live *M. fermentans* on MAPKs in general was not thoroughly studied. Most of the studies regarding *M. fermentans* and MAPKs were performed using mycoplasmal products or heat inactivated Mycoplasma (HIM). However, a few studies showed that infection of cells with various Mycoplasma can lead to ERK-phosphorylation [11], [17], [30]. In this study we examined the effect of MEK inhibitor PD-98059 on Topo I activity, alone or prior to infection with *M. fermentans*. MCF7 and U-251 cells were pre-incubated with PD for 1.5 hrs at a concentration of 25 µM followed by *M. fermentans* infection (MOI of 10^3^CFU/cell) and incubation for an additional 6 hrs. Nuclear extract was prepared and analyzed for Topo I activity and phosphorylation of ERK using Western blot analysis with specific anti p-ERK 1/2 antibody. The results demonstrated that the addition of MEK inhibitor to the Topo I reaction did not affect Topo I activity (Figure 6A, B), and in contrast, treatment of cells with the inhibitor (PD) prevented the *M. fermentans-*induced reduction in Topo I activity (Figure 3A,B for U-251 cells). In addition, we found that the quantity of p-ERK1/2 increased in infected cells (Figure 6C for MCF7 cells and Figure S3 C for U-251) while pre-treatment with PD prevented this increase. The level of total ERK was not affected by the various treatments (Figure 6C). These results suggest that the reduction in Topo I activity in *M. fermentans-*infected cells is mediated by MAPK signaling.

### 6. *M. fermentans* Infection Increased Poly-ADP-ribosylation of Topo I Derived From MCF7 Cells

**Figure 7:**
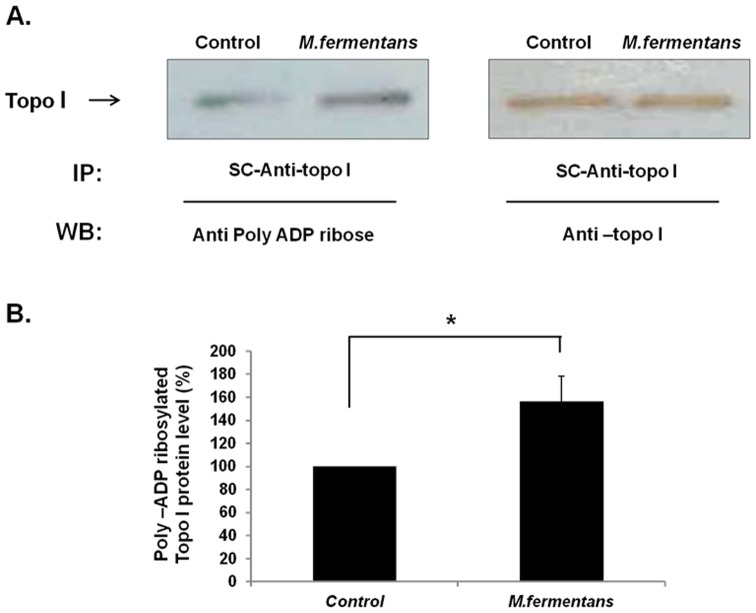
*M.* *fermentans* infection increased Poly-ADP-ribosylation (PAR) of Topo I derived from MCF7 cells. Nuclear protein extracts (200 µg) derived from *M. fermentans* infected and uninfected cells were subjected to immunoprecipitation with SC-anti-Topo I antibody. (**A**) The immuno complexes were analyzed by Western blotting with either anti-Topo I or anti-PAR antibodies. (**B**) Quantification analysis of Poly ADP ribosylated Topo I derived from MCF7 extracts. *Indicates *t*-test value of *p*<0.05.

PARP-1 regulates Topo I activity by ADP-ribosylation of the enzyme protein [46]. To examine whether infection with *M. fermentans* induced Topo I modification by PARP, Topo I protein was immunoprecipitated by anti-Topo I antibodies derived from scleroderma (SC) patient serum [47] and analyzed by Western blot using anti-Poly-ADP-ribose monoclonal antibody (Figure 7A). A protein of 100 kDa was immunoprecipitated by anti-Topo I antibody (SC serum), and reacted (in Western blot assay) with anti-Poly-ADP-ribose antibody (Figure 7A-left panel) and with goat polyclonal IgG anti-Topo I antibody, confirming the identification of the ADP-ribosylated Topo I. An increase in the level of Poly-ADP-ribosylation of Topo I in the *M. fermentans-*infected MCF7 cells (up to 160±20%, *p*<0.05) was observed in comparison to the uninfected cells (Figure 7B, n = 3 independent experiments).

### 7. Infection of Tumor Cells with *M. fermentans* Prior to CPT Treatment Reduced the Anti-cancer Efficacy of CPT

**Figure 8:**
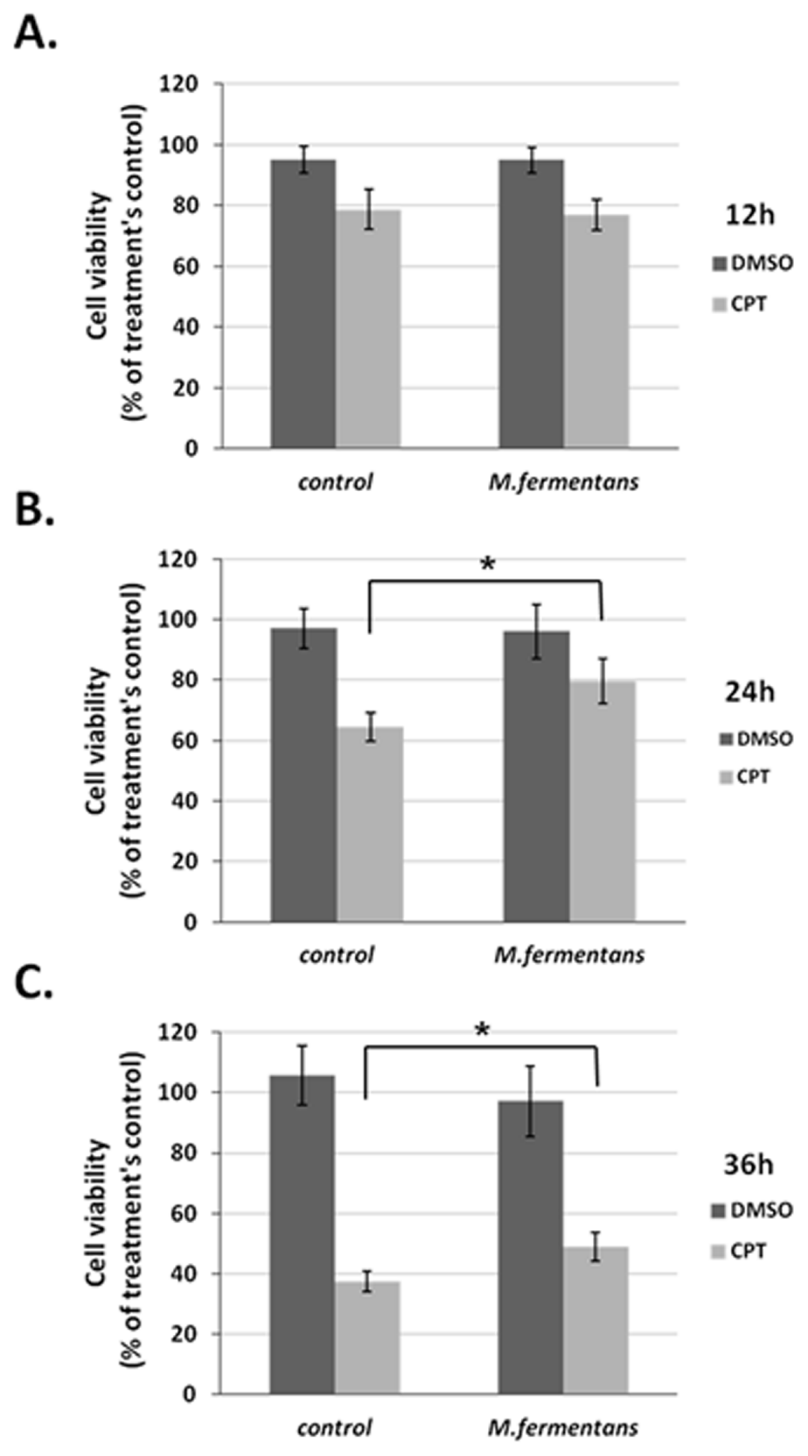
Infection of MCF7 cells with *M.* *fermentans* prior to CPT treatment altered the cytotoxic effect of CPT. MCF7 cells (5000/well) were infected with *M. fermentans* (MOI 2×10^3^) for 6 hrs followed by CPT treatment for 12 (A), 24 (B), or 36 (C) hrs. Cell cytotoxicity was examined by the Neutral Red assay. The Y-axis demonstrates the percent of cell survival calculated as follows: [%cell survival in Mycoplasma+CPT (or DMSO) treatment/% cell survival with Mycoplasma alone] ×100. The results are mean±SD of 3 independent experiments. *t*-test, *p<0.05.

MCF7 cells were infected with *M. fermentans* for 6 hrs at MOI of 2×10^3^ followed by CPT (30 µM) treatment for 12, 24, and 36 hrs. Cell viability was examined and the percent of cell viability relative to the treatment’s control was calculated. As described in Figure 8, pretreatment of the cells with Mycoplasma significantly decreased the cytotoxic level of CPT. An increase of 24±7.3% and 31±4.7% in cell survival (compared to the appropriate control of Mycoplasma-infected cells) was established 24 and 36 hrs, respectively, after CPT treatment. The results suggest that the infection of tumor cells with Mycoplasma altered the anti-cancer efficacy of CPT.

## Discussion

The efficacy of an anti-cancer drug depends on many cellular and environmental factors, of which a crucial one is the functionality of the target of the drug. Since Mycoplasma infect cells, including tumor cells, and this infection was previously shown to modify cell signaling [11]–[13], [17], we examined the influence of *M. fermentans* strain K7 on an essential anti-cancer drug target, the cellular DNA Topoisomerase I, and the effect of this infection on its inhibitor Camptothecin (CPT), the derivatives of which are currently in clinical use as anti-cancer drugs.

The results show, for the first time, that infection with live *M. fermentans* of breast cancer (MCF7) and glioblastoma brain tumor (U-251) cell lines significantly reduced (by 80%) the activity of cellular Topo I. This reduction was also observed when non-live (sonicated) Mycoplasma was used instead of live Mycoplasma, suggesting that the effect on cellular Topo I is probably mediated by Mycoplasma surface proteins/glycoproteins. The reduction in Topo I activity was detected 1.5 hrs after cell infection with *M. fermentans,* suggesting that Topo I is modified at early stages of the infection. The maximum reduction in Topo I (80%) was detected 6 hrs after infection, and a significant decrease in the enzyme activity (40%) was still observed 24 hrs after infection, indicating continuous modifications of Topo I activity in the presence of live or sonicated Mycoplasma. To understand the mechanism by which Mycoplasma infection reduced Topo I activity, several parameters were examined. First we showed that the reduction in Topo I DNA relaxation activity was not a consequence of a decrease in the enzyme protein level; thus, *M. fermentans* infection did not alter the expression of Topo I. Second, the effect of Mycoplasma infection on Topo I inhibition by CPT was examined. The results indicate that the level of inhibition of the Topo I DNA relaxation activity was reduced in infected cells by 4-fold compared to CPT-treated uninfected cells. Depletion in the free Topo I enzyme in the nucleoplasm of CPT-treated cells is a known phenomenon of CPT inhibition, which is a consequence of CPT-induced stabilization of the cleavable DNA-enzyme complex [21], [24], [25]. In Mycoplasma-infected cells, this Topo I depletion was partially diminished, implying that the enzyme protein was modified in the infected cells and this modification affected the enzyme-DNA interaction. Since infection with *M. fermentans* reduced the activity of cellular Topo I without affecting its protein level, it is reasonable to propose that *M. fermentans* infection of the tumor cells caused post-translational modifications of Topo I protein, reducing its activity. Topo I protein is known to undergo several post-translational modifications that influence its activity, such as phosphorylation of serine threonine residues, which is essential for its DNA relaxation activity, while dephosphorylation will reduce it. ADP-ribosylation of Topo I protein by PARP-1, as well as phosphorylation of tyrosine residues in the active site of the enzyme, are known to inhibit the DNA relaxation activity of Topo I [20], [27]. GlcNacylation of the enzyme protein was shown by us to affect the DNA relaxation activity of Topo I [26]. Therefore, to elucidate which of the aforementioned modifications may occur in *M. fermentans*-infected cells, we first examined the modification that reduced Topo I activity, namely ADP-ribosylation of the enzyme protein by PARP-1. The results revealed that treatment of cells with PARP-1 inhibitors prior to Mycoplasma infection totally diminished the Mycoplasma-induced reduction in Topo I DNA relaxation activity, suggesting that PARP-1 activation is involved in this process. To examine if Mycoplasma infection indeed induced PARP-1 activation, we looked for relevant signal transduction pathways that are induced in cells following Mycoplasma infection. It was previously shown that various MAPKs (i.e., SAPK/JNK, p38, NF-kB, AP-1, and ERK 1/2) were activated following the treatment of cells with lipid-associated Mycoplasma proteins (LAMP) [11], [29]–[31].

Unrelated to Mycoplasma infection, it was shown that PARP-1 is activated by ERK and its activity is up-regulated by ERK-catalyzed phosphorylation [28]. In this study, we have demonstrated that infection of cells with *M. fermentans* induced the phosphorylation of ERK1/2, which was prevented when MEK inhibitor was administered prior to cell infection. The notion that the decrease in Topo I activity in Mycoplasma-infected cells is mediated by the ERK signaling pathway was also demonstrated, since pre-incubation of the cells with MEK inhibitor prevented the Mycoplasma-induced decrease in Topo I activity. Moreover, we also demonstrated that in Mycoplasma-infected cells an increase in ADP-ribosylation of Topo I protein was observed.

Since Topo I is the target of the camptothecins, which are potent anti-cancer agents, the enzyme modification and the reduction in its activity may influence the efficacy of this anti-cancer drug in tumor cells that are infected with Mycoplasma. Indeed, we demonstrate that infection of MCF7 cells with *M. fermentans* prior to CPT treatment significantly decreased the cytotoxic effect of CPT. However, since we previously showed that CPT inhibited Mycoplasma growth {Horowitz, 1997 #55}, it is also possible that the alteration in CPT efficacy is also due to the consumption of CPT by Mycoplasma.

**Figure 9:**
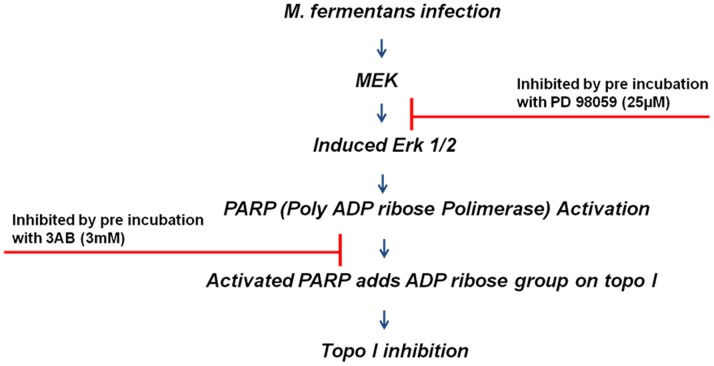
Proposed pathway that leads to the reduction of Topo I activity in M. fermentans-infected cells.

In summary, we believe that the pathway by which infection of cells with *M*. *fermentans* decreases the DNA relaxation activity of Topo I is via the induction of the MAPK signaling pathway in which ERK1/2 is phosphorylated by MEK. As illustrated in Figure 9, the p-ERK activates PARP-1, which modifies Topo I protein by ADP-ribosylation, decreasing the ability of Topoisomerase I to relax supercoiled DNA. Our data also suggest that this ADP ribosylation of Topo I interfered with its ability to bind DNA as demonstrated by the diminished inhibition of Topo I by CPT treatment in Mycoplasma-infected cells.

In conclusion, infection of tumor cells with Mycoplasma induced signal transduction pathways (i.e., the MAPK pathway including ERK2 and PARP-1) that can cause modifications of essential cellular enzymes such as Topo I and affect their activity. Moreover, the enzyme modification and the reduction in its activity influence the efficacy of its inhibitor as an anti-cancer drug. It is not yet clear if Mycoplasma infection of tumors occurs *in vivo* in patients, but our data clearly indicate that this possibility should be considered, specifically when anti-Topo I drugs are administered.

## Supporting Information

Figure S1 **M.fermentans diminished the CPT inhibition effect on the DNA relaxation activity of topo I.** U251 cells were infected with *M.fermentans* (*M.f*) for 6 hrs., followed by CPT (30 µM) treatments for additional 1.5 hrs. Total nuclear protein (12.5 ng) was added to a specific reaction mixture for Topo I. Reaction products were analyzed by agarose gel electrophoresis (**A**) a representative picture n = 3, of TopoI DNA-relaxation activity. (**B**) Quantification analysis of TopoI activity. Symbols: R and S are the relaxed and supercoiled form of the pUC19 DNA respectively, Topo- :no protein added to the reaction mixture. t-test: *p<0.05, **p<0.01, ***p<0.005
(TIF)

Figure S2 **PARP inhibitor prevented the mycoplasma- induced inhibitory effect on topo I activity.** U251 cells were pre -incubated with 3-aminobenzamide (3AB) for 1 hour at various concentrations followed by *M.fermentans* (*M.f*) infection (MOI of 10^3^CFU/cell) for additional 6 hrs. Total nuclear protein (12.5 ng) was added to a specific reaction mixture for Topo I. Reaction products were analyzed by agarose gel electrophoresis (A) and quantification of topo I activity was performed (B). Symbols: R and S are the relaxed and supercoiled form of the pUC19 DNA respectively, Topo- :no protein added to the reaction mixture. t-test: *p<0.05, **p<0.01, ***p<0.005
(TIF)

Figure S3 **M.fermentans induced ERK 1/2 phosphorylation and MEK inhibitor prevented the mycoplasma induced reduction in Topo I activity.** U251 cells were pre incubated with MEK inhibitors (PD) for 1 hour at concentration of 25 µM followed by *M.fermentans* (*M.f*) infection (MOI of 10^3^CFU/cell) for additional 6 hrs. Total nuclear proteins (12.5 ng) were added to a specific reaction mixture for topo I. Reaction products were analyzed by agarose gel electrophoresis (A) and quantification of topo I activity was performed (B). Phosphorylated ERK 1/2 protein level fromU251-extract was examined by Western blot analysis(C). Symbols: R and S are the relaxed and supercoiled form of the pUC19 DNA respectively, Topo- :no protein added to the reaction mixture. t-test: *p<0.05, **p<0.01, ***p<0.005
(TIF)
