# Supplementary figures and images for: Mycoplasma fermentans Inhibits the Activity of Cellular DNA Topoisomerase I by Activation of PARP1 and Alters the Efficacy of Its Anti-Cancer Inhibitor
Source: PLoS One. 2013 Aug 27;8(8):e72377. doi: 10.1371/journal.pone.0072377 (PMC3754970; doi:10.1371/journal.pone.0072377)

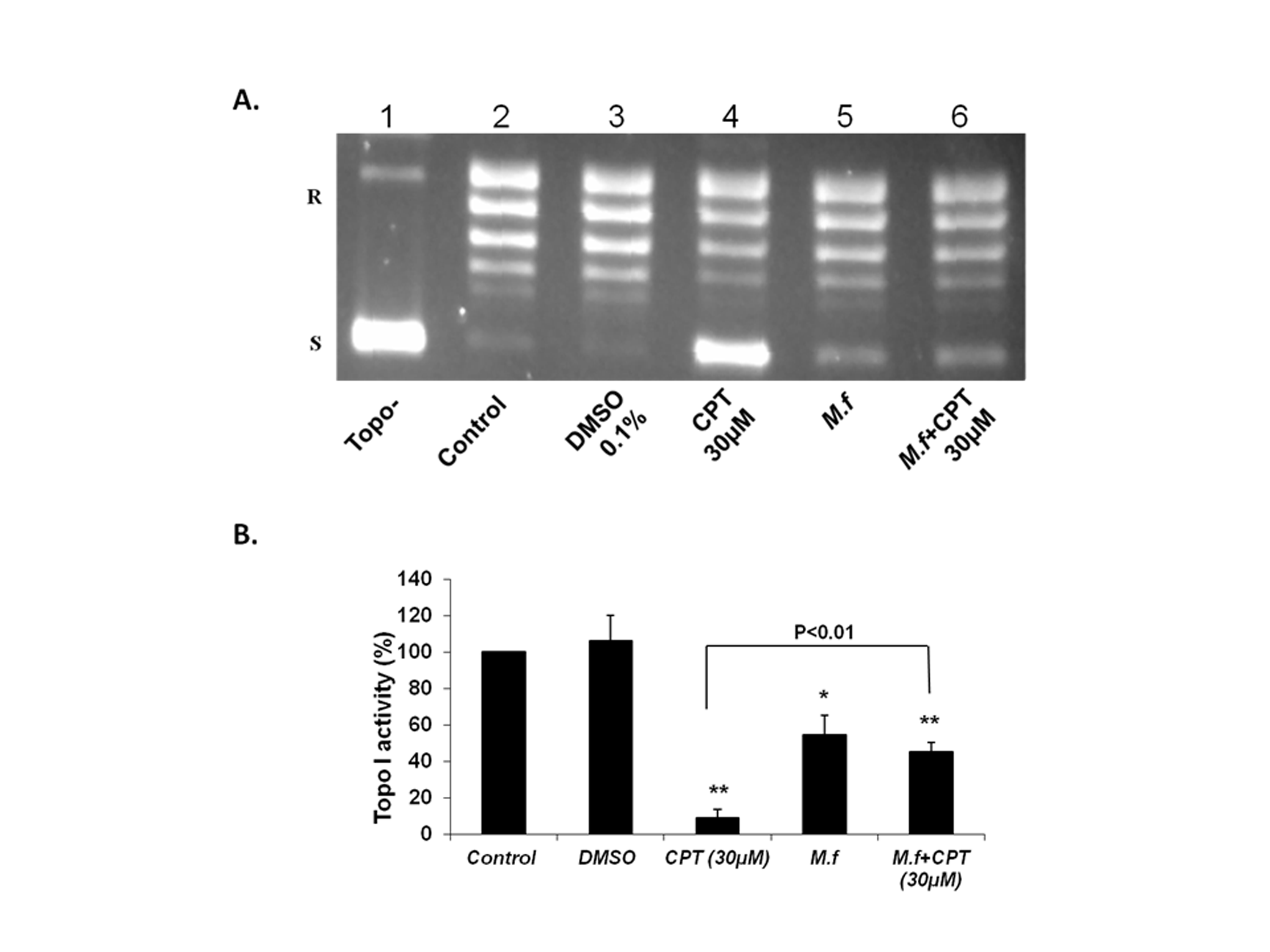

Supplement: Figure S1 — M.fermentans diminished the CPT inhibition effect on the DNA relaxation activity of topo I. U251 cells were infected with M.fermentans (M.f) for 6 hrs., followed by CPT (30 µM) treatments for additional 1.5 hrs. Total nuclear protein (12.5 ng) was added to a specific reaction mixture for Topo I. Reaction products were analyzed by agarose gel electrophoresis (A) a representative picture n = 3, of TopoI DNA-relaxation activity. (B) Quantification analysis of TopoI activity. Symbols: R and S are the relaxed and supercoiled form of the pUC19 DNA respectively, Topo- :no protein added to the reaction mixture. t-test: *p<0.05, **p<0.01, ***p<0.005 (TIF) [file pone.0072377.s001.tif]

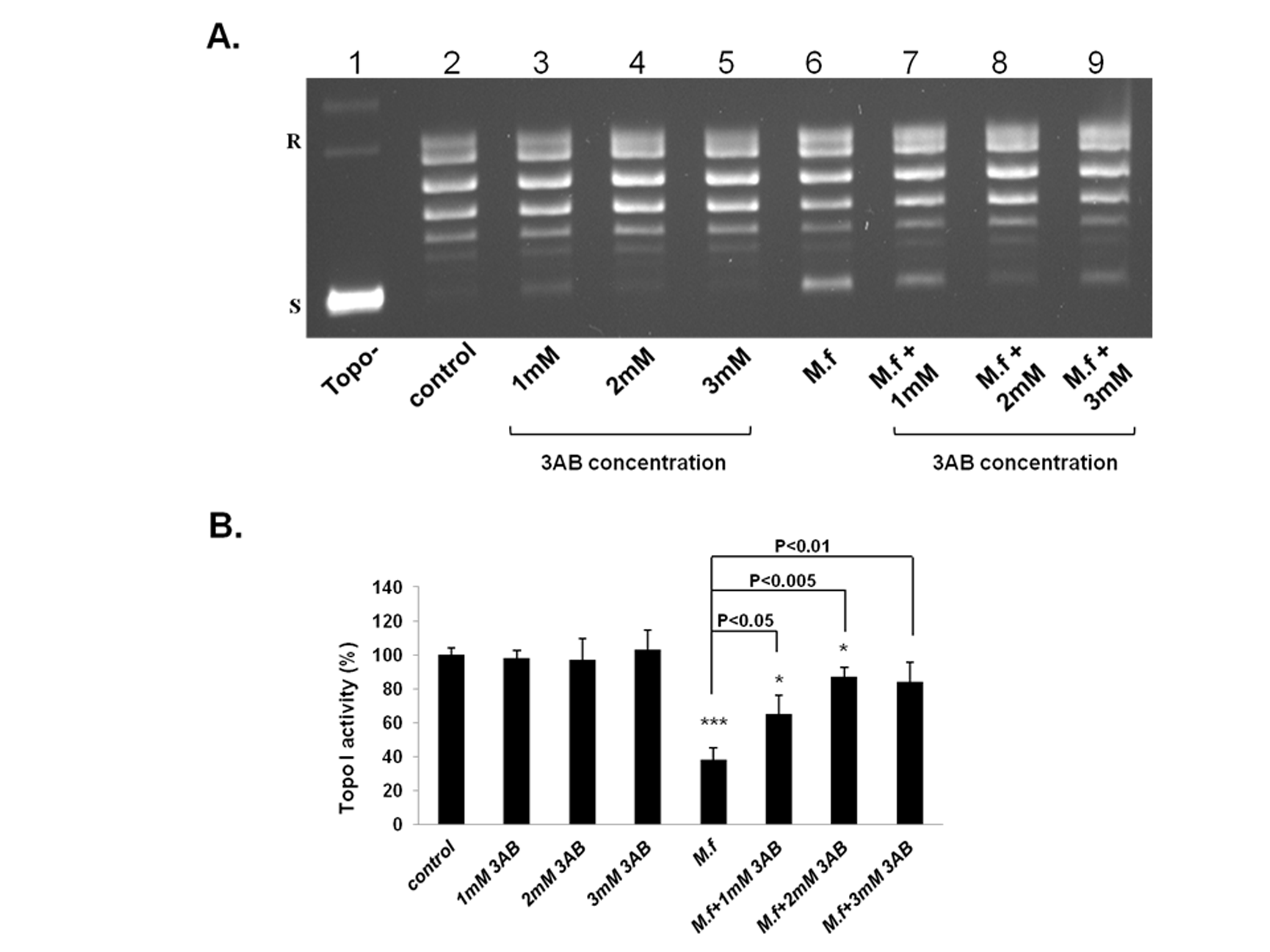

Supplement: Figure S2 — PARP inhibitor prevented the mycoplasma- induced inhibitory effect on topo I activity. U251 cells were pre -incubated with 3-aminobenzamide (3AB) for 1 hour at various concentrations followed by M.fermentans (M.f) infection (MOI of 103CFU/cell) for additional 6 hrs. Total nuclear protein (12.5 ng) was added to a specific reaction mixture for Topo I. Reaction products were analyzed by agarose gel electrophoresis (A) and quantification of topo I activity was performed (B). Symbols: R and S are the relaxed and supercoiled form of the pUC19 DNA respectively, Topo- :no protein added to the reaction mixture. t-test: *p<0.05, **p<0.01, ***p<0.005 (TIF) [file pone.0072377.s002.tif]

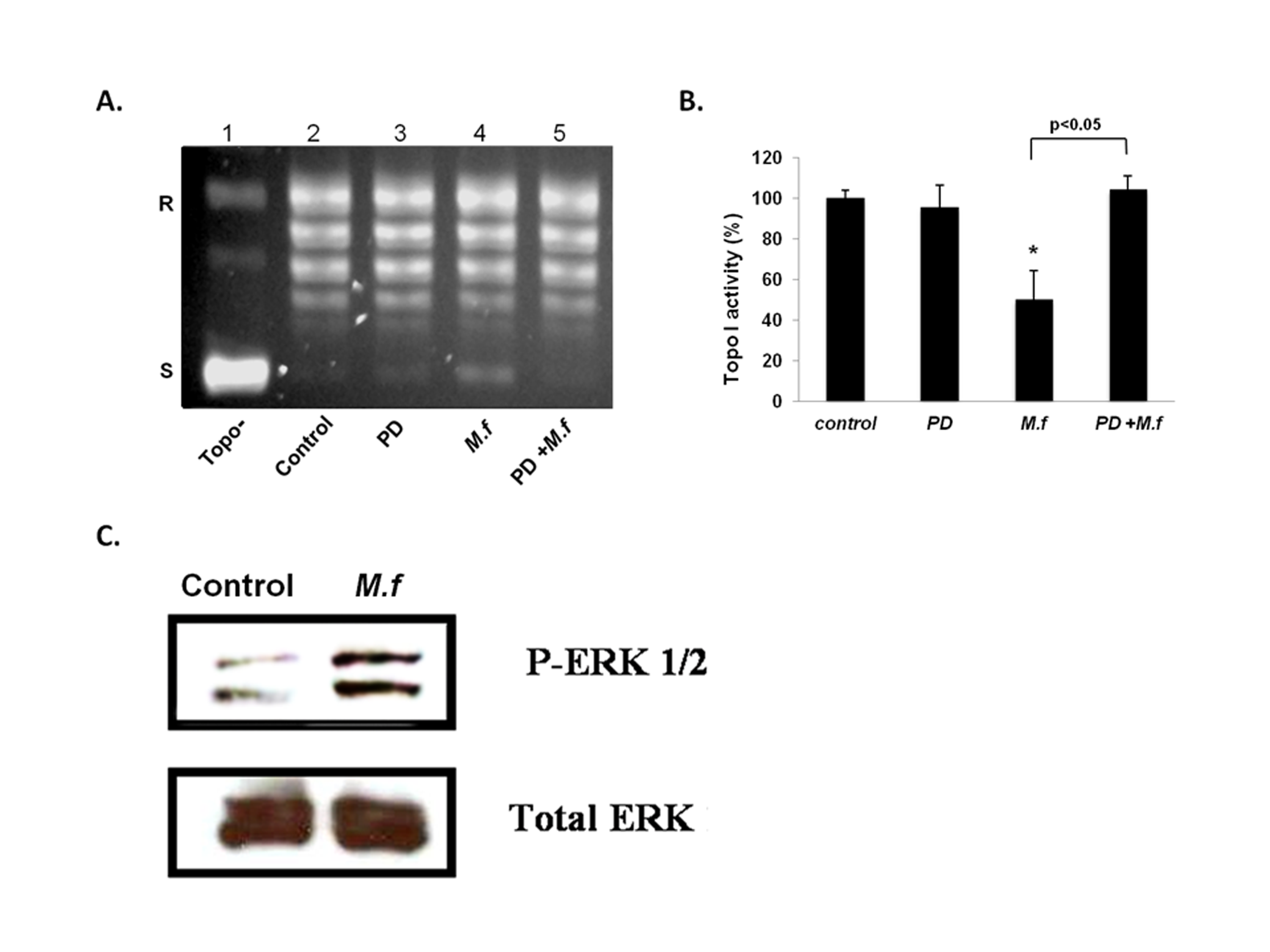

Supplement: Figure S3 — M.fermentans induced ERK 1/2 phosphorylation and MEK inhibitor prevented the mycoplasma induced reduction in Topo I activity. U251 cells were pre incubated with MEK inhibitors (PD) for 1 hour at concentration of 25 µM followed by M.fermentans (M.f) infection (MOI of 103CFU/cell) for additional 6 hrs. Total nuclear proteins (12.5 ng) were added to a specific reaction mixture for topo I. Reaction products were analyzed by agarose gel electrophoresis (A) and quantification of topo I activity was performed (B). Phosphorylated ERK 1/2 protein level fromU251-extract was examined by Western blot analysis(C). Symbols: R and S are the relaxed and supercoiled form of the pUC19 DNA respectively, Topo- :no protein added to the reaction mixture. t-test: *p<0.05, **p<0.01, ***p<0.005 (TIF) [file pone.0072377.s003.tif]
